# Case of Puerperal Fever, Followed by Phlegmasia Alba Dolens

**Published:** 1882-09

**Authors:** E. W. Berridge

**Affiliations:** London; Bureau Clinical Medicine, I. H. A.


### CASE OF PUERPERAL FEVER, FOLLOWED BY PHLEGMASIA ALBA DOLENS.

E. W. Berridge, M. D., London.

(*Bureau Clinical Medicine, I. H. A.*)

Mrs. ——, aged about 26, was delivered of her first child on October 23d, 1881. The labor was severe and prolonged, and I had to apply the forceps under chloroform. There was an extensive, though not deep, laceration of the perineum, which healed without sutures. I have had two other cases of lacerated perineum in primiparæ, one a forceps case; in all they healed up well by granulation, without the use of sutures or local medication. Of course, the sphincter ani remained intact, otherwise an operation would have been unavoidable. One of these patients, after the laceration had healed, was free from the pain of coition, which she had formerly experienced; she subsequently had twins without much trouble, proving that an artificial perineum may be better than the orginal one. In the treatment of this case I had the valuable assistance of a nurse, who not only understood her own department, but also was thoroughly versed in Homœopathy. I am indebted to her for much of the completeness of the following daily record. After the labor she complained of stiffness all over her, for which she took two doses of *Arnica* and subsequently *Nux* for ineffectual urging to stool, with spasmodic pain in rectum, coming on with forcing, like labor-pains, which the medicine relieved.

Oct. 25th. At 1 A. M., she had a rigor, lasting five minutes, but the pulse was only 78 (carefully counted four times by the nurse). She then slept till 5 A. M. All the morning was a little exhilarated, noticing and talking about things. At 1 P. M. the nurse noticed a slight odor about the patient and gave an enema of warm water, which brought away a very offensive large black clot; after which she gave an enema of Condy's Fluid. In thirty minutes more there was pain in uterus and shivering; she felt "as if all her inside was coming away," and an enormous quantity of nearly black clots passed from her, mixed with a little fluid blood; she shivered violently, and complained of feeling very cold. The nurse now gave her *Aconite*$^{mm}$ (Fincke), every fifteen minutes, for three doses, covered her up warmly, and put a hot-water bottle to feet. The shivering now ceased and she perspired; the pulse, which before the *Acon.*

was 118, had fallen in an hour to 108. She then slept, the pains being better. In another hour the shivering returned, and the pulse rose to 120. The nurse repeated the *Acon.* every five minutes, for eight doses.

I saw her at 4 P. M.; she was quiet; no shivering; pulse, 120; perspiring; lochia scanty, *offensive black clots* mixed with water; there had been signs of the milk the night before, but now the breasts were very flabby. The italicized symptoms pointed to *Kreosotum*, according to C. Lippe's most excellent *Repertory*, and I ordered a dose of 1M (Jenichen) every two hours, till better.

At 11 P. M., she felt much better; lochia freer; pulse, 120. Had taken two doses, and subsequently took one at midnight and again at 4. A M.

Oct. 26th, 9. A. M. Slept well last night. Pulse is now 96; less pain in uterus; lochia more natural in consistency, more red, less fetid. No medicine.

2.30 P. M. Soon after my last visit, had pains over right eye; then slept for a long time and was free from pain, but pulse rose to 108, and lochia became very *offensive*, *dark*, with a few black strings, but no clots; *no milk;* very slight tenderness of uterus; temperature, 101.8. *Bellad.*$^{60m}$ (Fincke), every two hours for three doses.

10 P. M. Pulse, 108; temperature, 99.6; lochia more free, very offensive; natural stool; milk returning; uterus tender only on hard pressure. No medicine.

Oct. 27th. Slept well from 1 A. M. to 3 A. M., when she awoke with pain in left calf, which, however, she did not mention till 5 A. M.; lochia continue free. The nurse gave her another dose of *Bell.* and applied a wet bandage to calf, which relieved. At 10 A. M., I saw her; there was a deep-seated hard lump in left calf, very tender, with the integuments freely movable over it; temperature, 99.4; milk disappeared; pulse, 120, irregular in volume; heaviness in left lower leg; at first the pains made her move her leg constantly; now it is painful only when moved. Under "Swelling in Calf," Lippe's *Repertory* gives *Bry.*, *Chin.*, *Mezer.*, *Puls.* The pain compelling the patient to move the limb indicated, the last medicine (compare Dr. Walter M. James' case in *The Organon*, vol. iii, p. 34), *Pulsat.*$^{cm}$ (Fincke), one dose.

At 3 P. M., I saw her again; she had improved in half an hour after the dose, the breasts became fuller, and the child fed without causing much pain. Bowels have acted naturally. Leg still swelled, with heaviness, but less pain on moving. Pulse, 114; temperature, 101.7. Lochia more natural; less dark fluid, but rather thick, and still offensive. The nurse has given her another injection of Condy's Fluid. Feet cold and damp. The odor seems to proceed not only from the lochia, but from the entire body. I gave another dose of *Pulsatilla*$^{cm}$ (Fincke).

At 9.15 P. M., pulse was 120 and temperature 100.8, lochia less offensive and more natural; milk coming in slowly; leg unchanged. The nurse repeated the *Pulsat.* half an hour ago.

Oct. 28th, 9.45 A. M. Slept well till 5 A. M.; leg still heavy, but swelling less, and much less tenderness; lochia of better color, and much less offensive; more milk; pulse, 100, stronger; temperature, 98.4. Has taken no more medicine.

At 9 P. M., visited her; about 4 P. M. she had had some chilliness, but not a rigor; previously the leg had been rather more painful, and about 6 P. M. decidedly so, with hard swelling extending all over calf. The nurse repeated the *Pulsat.* Now leg is less painful, and the swelling softer and less extensive, but the lump is still more tender than this morning. The lochia are unchanged in character, but much freer, and *make her feel faint as they flow.* (The nurse tells me that years ago, she verified this italicized symptom with *Pulsat.*$^{30}$, and that she has also cured with it the same symptom, occurring during the otherwise natural menses.) Milk is less plentiful. No abnormal uterine tenderness to-day. Pulse, 106; temperature, 99. Since the last dose, there has been less faintness from the flow of lochia. From 4 P. M. to 6.30 P. M., she seemed faint and low, scarcely speaking above her breath.

Oct. 29th, 10 A. M. Has not slept so well; lochia more scanty, more coffee-colored, and still a little offensive. Natural stool this morning. Leg much better, lump smaller, with less pain and tenderness. Pulse, 108; temperature, 99. Milk less free. *Pulsat.*$^{mm}$ (Fincke), one dose.

At 9 P. M., I saw her; the nurse had repeated the *Pulsat.* at 2, 4 and 6 P. M. She seemed poorly all day, restless, inclined to cry, with distaste for food, except of a liquid nature. The leg became painful at 6 P. M. (about the same time as yesterday). It had been better all day, but she was not so well in herself; when the leg became worse, she felt generally better. This evening she slept well, but since the morning till this evening, she kept dozing, with absurd dreams, which are quite unusual. During this evening's sleep, there was warm sweat over whole body, especially in legs. Lochia slightly offensive, otherwise unchanged. Breasts more flabby. Pulse, 88; temperature, 98.2. Swelling in calf unchanged.

The action of *Pulsat.* had evidently ceased, and new symptoms had arisen. "Sweat during sleep," is given in Lippe's *Repertory*, under *Carb. an.*, *Cic.*, *Chin.*, *Dros.*, *Euphorb.*, *Ferr.*, *Jatr.*, *Merc.*, *Nux*, *Phosp.*, *Puls.*, *Selen.*, *Thuya.* Of these, only *China* and *Puls.* have the swelling of the calf; and the former medicine was also indicated by the absurd dreams and the periodicity of the pain in the leg. I gave one dose of *China*$^{cm}$ (Fincke).

Oct. 30th, 11 A. M. Pulse, 96; temperature, 98.8. Slept well. Lochia ceased all night, but returned this morning. No sweat; much less dreaming, and only when dozing—not during sound sleep; no uterine tenderness; more milk; ineffectual urging to stool this morning, for the first time since the second day; lump in calf unchanged, no swelling around it; appetite much better; is in good spirits. This morning, asked to have the blinds up, hitherto has desired the room to be dark.

At 9 P. M., pulse, 94, and stronger; temperature, 98.4. Lochia returning, brighter, and without odor; no sweat; slept without dreaming; no uterine pain; the pain in the leg did not return, as before, at 6 P. M., otherwise it is unchanged; appetite good; much stronger; natural stool; laceration in perineum healing; milk more scanty this evening, and causes diarrhœa in the child.

Oct. 31st, 9.40 A. M. Had restless and troubled sleep up to 2 A. M., when she woke, feeling very hot, with much sweat. The nurse gave a dose of *China*$^{cm}$ (Fincke). After this she slept better, and at 5 A. M. felt much better and had a good breakfast. No more absurd dreams. This morning she feels stronger and her voice is stronger. The lump in calf is not so clearly defined; the surrounding integuments are swollen, but soft; very little pain in it. Not much milk; has never felt it flow in naturally; it still produces diarrhœa and rumbling in the child as soon as taken. Lochia nearly ceased, very slightly offensive. Pulse, 96; temperature, 98.8.

At 8.30 P. M., pulse, 96; temperature, 98.8. Swelling much less and lump much smaller; no increase of pain at 6 P. M. Lochia returned freely and appear natural. She has twice (for the first time) felt the draught of milk naturally; it is more free and no longer purges the child, and he sleeps after it. No stool to-day. Is stronger and pulse stronger.

Nov. 1st, 9.30 A. M. Went to sleep at 10.30 P. M. yesterday, and at 11.30 P. M. was moaning, so that the nurse woke her; she said she had had nightmare and was restless and feverish. The *China* was repeated, and she soon fell asleep till 3.30 A. M., when she awoke hungry. Lochia natural. Draught of milk felt. It is morning. Last night at 9.30 P. M., the milk caused diarrhœa in the child as soon as taken; this diarrhœa has always been green, the milk would go through him at once with a rattling noise in bowels. Pulse, 96; temperature, 98.8; leg improving.

At 8.45 P. M., pulse, 96; temperature, 98.8; milk more abundant and does not disagree with the child. This afternoon had absurd dreams and nightmare again. The leg has not troubled her. Is cheerful. The day is very cold and she easily gets cold, especially about the legs, and asks to be covered up. Repeated dose of *China*$^{cm}$ (Fincke), at 9.30 P. M.

Nov. 2d, 9.30 A. M. Last evening was restless and felt weak. The nurse repeated the *China* at 11 P. M.; soon afterward went to sleep and slept well till 3 A. M.; very little dreaming and could not remember her dreams. Lochia natural. The milk agrees with child. No sweats. Before her accouchement, there had been internal and external swelling of the sexual parts; this has now disappeared. The laceration of perineum is healing. Bowels are natural. Temperature, 99; pulse, 93; the lump in calf is much smaller and less tender, no pain in it.

Nov. 3d, 9. A. M. Pulse, 90; temperature, 98.2. Slept well; looks better; swelling in calf unchanged. Milk free, but *thin and blue,* and soon causes profuse urination in child, who literally swamps the bed. The patient herself urinates often, passing much at a time; it is pale, like weak green tea. When the child's bowels were acted on by the milk, the urine was not affected, and now it is *vice versa.* The italicized symptom pointed to *Lachesis* (C. Lippe's *Repertory,* p. 199), and I gave one dose of CM (Fincke).

Nov. 4th, 9 A. M. All yesterday was much better in every way, and in the evening the lump was much smaller. In the afternoon there was an escape of wind from vagina, which she never had before. Slept fairly, but the night was hot and oppressive. Milk much thicker since yesterday evening; child vomits some of it but has no diarrhœa. Pulse, 96; temperature, 99. The lump is the same as yesterday, not painful, except from hard pressure.

Nov. 5th, 9.30 A. M. *Pulse,* 96 (*was* 86 *last evening*). Temperature, 98.7. Last night after 9 P. M., had a fright from her husband's return home being delayed; she lay thinking over it, and at last lay with eyes starting, wide open and very bright; clutching at the bed-clothes and twitching all over. When her husband returned she lay speechless, with a stony, corpse-like look, except for the color in lips, and at length drew a long sigh and burst out crying, which relieved her. The nurse then gave her a dose of *Opium*$^{cm}$ (Swan), which quieted her, and she slept well. The child urinated less, yesterday, but last evening had distention and hardness of abdomen, with pain and crying, for which the nurse gave him a dose of *Lycop.*, with relief. The swelling of the calf is unchanged, but there is very little tenderness. Lochia natural. Milk plentiful, natural. Yesterday stools very large and difficult, causing straining.

The pulse was quicker in the morning than the evening, a very unusual symptom, which, according to C. Lippe's *Repertory* (p. 264), belongs to *Agar.*, *Arsen.*, *Kali c.*, *Sulph.* and *Thuya.* Of these I selected *Sulphur* as alone corresponding to the swelling of calf, and gave one dose of DM (F. C.).

Nov. 6th, noon. Lump smaller last evening and about the same to-day. Milk natural, but gives the child pain; child urinates less. Lochia natural; sleeps well; temperature, 98.6; pulse, 90. Tongue furred at back. For three or four days there has been sweat on the soles of the feet, no other part, and the soles are yellow; the sweat was very offensive yesterday in spite of daily washing. For two days no stool, with ineffectual urging. The sweat on the soles belongs (C. Lippe's *Repertory*, p. 228,) to *Acon.*, *Amm. mur.* and *Nitr. ac.* Of these the latter alone (see p. 135) has ineffectual urging. Had these symptoms been mentioned to me the previous day, I should have considered them as of more importance than those indicating *Sulphur*, because one of the *Sulphur* indications (the swelling of calf) was an old symptom, and therefore of less diagnostic value than the later ones. Therefore, as the *Sulphur* seemed to have effected nothing but the reduction of the swelling, and the new symptoms did not correspond with it, I did not wait its further action, but gave one dose of *Nitr. ac.*$^{mm}$ (Fincke).

Nov. 7th. The ineffectual urging continued increasing all the evening, so the nurse dissolved *Nitr. ac.*$^{mm}$ (Fincke), in water, and gave three doses during the night. This morning, natural stool, the first since Nov. 3d. To-day (3.30 P. M.), pulse was 84; milk natural, it does not cause any unnatural urination in the child (this improvement is marked since the *Nitr. ac.*), but it causes colic. The lump in calf is unchanged. Tongue still furred at the back. Sweat on soles much less, and less offensive. (The odor of the feet-sweat was something like the odor of a stable; the lochia had the odor of stinking fish.) Since her accouchement, had urinated too frequently, a copious amount each time; since the *Nitr. ac.*, this has improved; it was never so before. Soles yellow only in patches.

About 4 P. M. she had sudden, violent cutting pains in small of back, shifting to sides, abdomen, below scapulæ, etc., causing her to cry out and twist about in every position. I gave *Lycop.*$^{cm}$ (F. C.) every few minutes, but with only slight relief. I then gave *Kali carb.*$^{3cm}$ (Fincke), four doses. There was relief at once after the first dose; in fifteen minutes she took the second, and in twenty minutes more the third, as she felt the pain returning. The pain again returned about 6 P. M., and she took two doses in thirty minutes. At 10 P. M. I saw her again. She had no return of pain, but feels sore and very weak; very thirsty, craving champagne, which she has had. (She never takes stimulants when in health, nor has she hitherto craved for any during her illness.) The cause of this colic seemed to be that she had eaten some stewed celery. The nurse had eaten some, and had afterwards profuse noisy eructations and profuse flatus downwards. The child took the breast since its mother partook of the celery, since which it has lost the painful flatulence, and it now rolls up easily. Thus the celery removed in the infant symptoms similar to those which it produced in the mother.

Nov. 8th, 10.15 A. M. Took another dose of *Kali carb.* about 2 A. M., as she thought the pain was returning. Soon after my last visit, she had constant pain in small of back, as if strained, worse on moving; on lying on other side, pain in the corresponding hypochondrium like a dragging from the other side. Urine turbid and high-colored. Very restless last night, moaning loudly and hysterical; tongue creamy-white, coated as far as tip; breath offensive; ineffectual urging to stool; temperature, 98.5; since last night the lump has moved more to the inner side of calf, and is a little larger, spreading out more; no appetite; nausea to food; feels flatus in *chest* when lying, relieved by sitting up, when it rises; if it does not rise it obstructs the breathing (the latest symptom). Back relieved by lying on it; a new medicine was evidently indicated. The latest, and therefore the most important symptom, the obstruction of the breathing by flatulence, belongs to *Mezereum* (Lippe's *Repertory*, p. 119), and it has also the swelling of calf, which was now increasing again. I gave one dose of *Mezereum*$^{3m}$ (Jenichen).

At 9 P. M. I saw her again; she had taken another dose at 2 P. M. and 6 P. M., when the obstruction of breathing returned, but on the whole has been much better. Now the pain in back, and the pain in side, when lying on it, is much better; urine less dark and thick, but became very much higher-colored and turbid after the first dose of *Mezereum*. No stool or urging; tongue clearing toward tip; has slept two hours; is quieter altogether; breath less offensive; lump in calf smaller; no appetite; still nausea to food.

Nov. 9th, 3.30 P. M. About 1.30 A. M. felt some return of pain in back and took another dose. Now has no pain in sides, and very little in back. Tongue clearer. Since the morning, jaundiced all over. Urine less high-colored and less thick. Bowels relieved; stools large, all in one or two pieces, which were knotty in shape and difficult; milk natural; lochia returning, a little bright, without odor; feet-sweat nearly gone, and not offensive; flatulence very slight; appetite poor; slept well and awoke refreshed; breath slightly offensive; lump in calf unchanged; temperature, 98.7; pulse, 78. Yesterday afternoon, the right side of abdomen was larger than the left; this has ceased since the last evacuation. As the patient was generally better, I gave no more medicine, notwithstanding the occurrence of the jaundice; the result proved that I was right.

Nov. 10th, 3.45 A. M. Slept soundly from midnight to 6 A. M. Much less jaundice; urine more natural, but still high-colored and strong-smelling, only turbid after standing; no stool; some swelling in right abdomen; lump in calf much softer; sticky sweat all over for two days; sweat on soles very slightly offensive; tongue still coated at back; pain in back much better; pain in hypochondrium quite gone. Is sitting up, but feels very weak, and has to sit bent forward. I left her a dose of *Phosphorus*$^{cm}$ (Fincke), in case the sticky sweat continued (see Lippe's *Repertory*, p. 257).

Nov. 11th. Sweat was very sticky last evening, so she took the dose of *Phosph.* about 11 P. M. To-day, sweats decidedly less, and less sticky. Dreamed of being pursued last night (effect of *Phosph.*). Less swelling of calf; less jaundice; natural stool; milk disappearing, it gives the child colic if he takes it more than once a day, and the first time it gives him wind; urine more natural; tongue clearer; stronger; pain in back and side gone; appetite returning; for four days has felt hungry soon after food.

Nov. 12th. Took another dose of *Phosph.* last night, as there was still sweat. Had a good night; sweat now gone; tongue clearer. Appetite better, but feels hungry very soon after eating; eructations after food. Swelling of calf unchanged. A little hysteria to-day, wanted to cry. Milk returned to-day; also, the lochia, which are bright red; no stool. In Lippe's *Repertory*, p. 158, I found: "Lochia turning bloody again, *Cauloph.*, *Rhus*, *Secale*." Of these three, the latter alone has "irresistible hunger" (p. 88), so I gave *Secale*$^{cm}$ (Swan).

Nov. 14th, 3 P. M. Had four doses of *Secale* yesterday; none to-day; less ravenous since first dose. Less swelling in calf this morning; has walked a little. A little sticky sweat. Still has eructations after food. "Feels the heart beating in the throat." Milk returning. Lochia very free and very bright, and she feels very weak. Tongue coated only at back. Is still a little yellow, but the natural color of skin is returning. *Secale*$^{cm}$ (Swan), twice a day.

Nov. 15th. About 6 or 7 P. M., yesterday, had another attack of pain in waist and abdomen, moving about; like the former attack, but less severe. She had been unpleasantly excited a short time previously. About 8.30 P. M., the nurse, who had been away, returned and gave *Kali carb*$^{cm}$ (F. C.), every five minutes, for an hour, till the pain was decidedly better, then every fifteen minutes; it soon commenced to give relief. Lochia as before, and milk returned freely in evening. Had feeling of great weakness and faint feeling for food. With this attack of pain, and also with the first one, had feeling of hot blood running down inside spine from between scapulæ to waist. I saw her to-day at 4 P. M.; she had had a bad night from restlessness and flatulent pain; she had taken several doses of *Kali* in night, which relieved, but not nearly so quickly as before. To-day, is much stronger; bowels natural; skin less yellow. Lochia and milk unchanged. Lump in calf nearly gone, and she has walked down-stairs; sweat; ravenous feeling and sensation of blood flowing down spine all gone. The milk now agrees with the child.

Nov. 17th. Had a little pain in back yesterday, and took two doses of *Kali;* also, one to-day, when the ravenous appetite returned. She still feels sinking, exhausted and faint about thirty minutes after food. Lochia still very free; milk natural, but nursing makes her feel hysterical and exhausted. Lump in calf almost gone, but there is a little dragging there on walking. Has been out for a drive and feels stronger. On account of the exhaustion from loss of fluids, with the other symptoms, I prescribed *Calc. carb.*$^{cm}$ (F. C.), twice a day.

Nov. 19th. On 17th, about 6 P. M., before she had taken the *Calc.*, she had a return of the flatulent pain. The nurse gave her two doses of *Calc.* and two of *Kali carb.*, but without relief. The patient then took some brandy and soda, without relief; followed by some peppermint, which brought up a little wind. The nurse then gave her two doses of *Carb. veg.*$^{cm}$ (F. C.); the first dose checked the pain from increasing, and the second removed it. The swelling in calf is unchanged to-day, with stiffness still on walking, especially on descending stairs. Lochia nearly gone. Had two doses of *Calc.* yesterday, and one to-day. The hunger after food is less. Less weakness after nursing, but still weak and inclined to cry after it. *Calc.*$^{107m}$ (Fincke), morning and evening.

Nov. 21st. Last night at 10 P. M. had another attack of flatulent pain, relieved as before by *Carb. veg.;* she took it every five minutes for eight or ten doses; relief commenced after sixth dose. Yesterday the ravenous appetite had ceased, so the nurse gave her no more *Calc.*, nor to-day either; has had two doses of *Carb. veg.* to-day. Lochia ceased. Lump in calf almost gone. Nursing does not affect her much now, but the milk does not satisfy the child. Only a little stiffness in both legs on going down-stairs. For three days, swelling of labia, uncomfortable on sitting. Feeling of lump in stomach after a full meal. Yellow fur at back of tongue. The swelling of labia is a symptom of *Carb. veg.* (see Lippe's *Repertory*, p. 160), and as the patient was better, I ordered it to be repeated only if the pain returned.

Nov. 23d. Had two doses of *Carb. veg.*$^{cm}$ (F. C.), on evening of 21st, because the pain returned; no medicine since. Better now in every way; not ravenous; feeling of lump in stomach gone; swelling in calf nearly gone; only a little stiffness in legs; swelling of labia much less; return of bright lochia, yesterday; only a little yellowness of skin; milk plentiful and exhausts her less. *Carb. veg.*$^{cm}$ (F. C.), once a day.

Nov. 25th. No return of flatulent pain; is much stronger; no swelling of labia; lump in calf unchanged, but leg rather more swollen around it. *Carb. veg.*$^{cm}$ (F. C.), once a day.

Dec. 2d. No more flatulent pain; leg natural, except a little difficulty in walking down-stairs, which she thinks is only from nervousness. On Nov. 25th and 27th, had a return of the flatus from vagina. Is much stronger.

Dec. 6th.—Quite well, except that the child's appetite has caused soreness of nipples.

